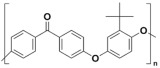# Recent Progress on the Development of Polyetheretherketone Membranes for Water Remediation

**DOI:** 10.3390/membranes15090256

**Published:** 2025-08-28

**Authors:** Jingwen Zhou, Longjun Wang, Hong Liu, Xinhao Li, Dalong Li, Linlin Yan, Xiquan Cheng

**Affiliations:** 1 State Key Laboratory of Urban Water Resource and Environment, School of Marine Science and Technology, Harbin Institute of Technology, Weihai 264209, China; 23s030055@stu.hit.edu.cn (J.Z.); 23S030089@stu.hit.edu.cn (L.W.); 2024210783@stu.hit.edu.cn (H.L.); 2023210571@stu.hit.edu.cn (X.L.); lidalong@hit.edu.cn (D.L.); 2 Shandong Sino-European Membrane Technology Research Institute Co., Ltd., Weihai Key Laboratory of Water Treatment and Membrane Technology, Weihai 264209, China

**Keywords:** PEEK, membrane preparation, hydrophilic modifications, water remediation applications

## Abstract

Industries containing excess acid or alkaline wastewater exacerbate water security. As a semi-crystalline engineering thermoplastic with superior chemical resistance, exceptional mechanical strength, and outstanding thermal stability, polyetheretherketone (PEEK) is a promising candidate for advanced functional membranes in water remediation. Herein, we present a comprehensive overview of recent advances in PEEK materials, encompassing PEEK membrane fabrication, strategies for membrane hydrophilic modification, and applications in wastewater treatment. Specifically, research efforts have focused on membrane preparation methods such as nonsolvent-induced phase separation (NIPS), thermally induced phase separation (TIPS), and chemical-induced crystallization (CIC), which aim to address the critical challenge of forming solvent-resistant PEEK membranes while maintaining membrane performance. Additionally, various hydrophilic modification strategies (pretreatment, co-blending, and post-treatment) for PEEK membranes are discussed to alleviate membrane fouling problems, with in-depth discussions of diverse applications in wastewater treatment (such as the removal and purification of synthetic dyes, organic solvents, natural organic matter removal, and oil–water mixture). The review concludes with an emphasis on the current challenges and potential of PEEK membrane for wastewater treatment.

## 1. Introduction

Membrane technology has grown rapidly as a leading green solution in recent decades, offering advantages including small occupation area, low energy consumption, operational simplicity, and compatibility with existing technologies [1,2]. It serves crucial roles in wastewater treatment such as industrial process optimization, pollution mitigation, and resource recycling, delivering significant economic and environmental benefits [3,4,5]. However, modern water treatment increasingly involves complex aquatic environments characterized by strong acids, alkalis, organic solvents, and elevated temperatures, which pose a challenge to traditional polymer membranes. The currently widely used materials, including polylactic acid (PLA), polyethersulfone (PES), polyacrylonitrile (PAN), and polyimide (PI), suffer from limited chemical/thermal stability and mechanical strength to withstand these harsh conditions [6,7]. For instance, PLA membranes undergo irreversible structural dissolution through pore collapse and polymer chain scission when exposed to aggressive organic solvents like N-methylpyrrolidone [8]. This underscores the urgent need for advanced membrane materials with superior corrosion resistance and thermal stability to meet practical industrial demands.

Polyetheretherketone (PEEK) has stepped out as a compelling candidate among them, uniquely integrating ceramic-like chemical and thermal stability with the flexibility, processability, and cost-effectiveness of polymers [9]. Specifically, PEEK is a semi-crystalline engineering thermoplastic characterized by a rigid aromatic backbone structure of alternating phenyl ether and benzophenone units [10], which endows the polymer with a suite of exceptional properties such as superior chemical resistance (encompassing ultraviolet radiation, strong acid, alkali, organic solvent, and high salt concentrations), exceptional mechanical strength (including tensile, compressive, and impact resistance), and outstanding thermal stability (retaining properties up to 250 °C). These attributes enable PEEK to serve as a high-performance matrix material for separation membranes, enhancing their durability in applications like organic mixture purification, heavy metal salt adsorption, and macromolecule/impurity filtration. Furthermore, these characteristics also underpin the adoption in aerospace components, biomedical applications, and petrochemical processes [11,12,13].

**Figure 1:**
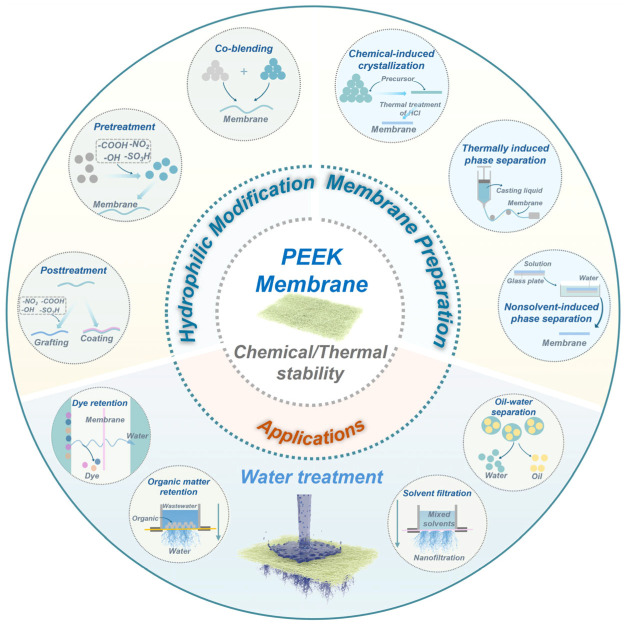
Membrane fabrication, hydrophilic modification strategies, and wastewater treatment applications for PEEK membranes (drawn by 3Dmax, Adobe Photoshop, and PowerPoint).

This review systematically synthesizes recent advancements in PEEK membranes, following a clear narrative flow centered on their development and application (Figure 1). Firstly, it outlines the key progress in PEEK membrane preparation, covering diverse fabrication methods such as thermally induced phase separation (TIPS), nonsolvent-induced phase separation (NIPS), or chemical-induced crystallization (CIC). Next, the review delves into three core hydrophilic modification strategies such as pre-fabrication chemical functionalization of the base polymer, co-blending modification, and post-fabrication surface grafting/surface coating, addressing PEEK’s intrinsic hydrophobicity to enhance performance such as permeance, selectivity, and antifouling capacity. It further summarizes the practical applications of the PEEK and modified PEEK membranes in water treatment scenarios encompassing dye separation, oil–water removal, organic solvent nanofiltration, and natural organic matter removal. Finally, the review synthesizes key progress made in the field, discusses the critical challenges that remain, and offers insights into future directions for advancing PEEK-based membranes as next-generation water treatment materials.

## 2. Preparation of PEEK Membranes

Polyetheretherketone (PEEK) is a semi-crystalline thermoplastic featuring a rigid aromatic backbone comprising alternating ether and ketone linkages, which confers high crystallinity and strong intermolecular interactions. The densely packed molecular chains form diffusion-barrier matrices that impede solvent permeation, endowing PEEK with remarkable chemical inertness in aggressive environments and reducing processability [14]. Specifically, the exceptional chemical stability of PEEK renders it insoluble in common organic solvents including N-methyl pyrrolidone (NMP), N, N-dimethylformamide (DMF), and dimethylacetamide (DMAc), posing serious challenges for membrane fabrication. This section systematically reviews dominant membrane fabrication techniques, with particular emphasis on Nonsolvent-induced phase separation (NIPS), Thermally induced phase separation (TIPS), and Chemical-induced crystallization (CIC) strategies.

### 2.1. Nonsolvent-Induced Phase Separation (NIPS)

**Figure 2:**
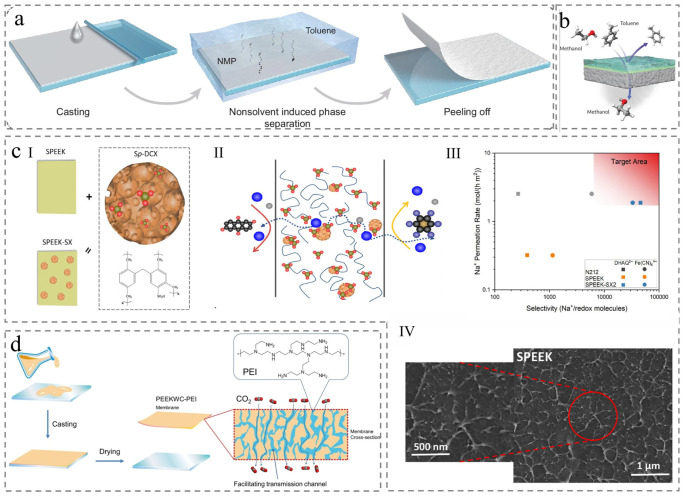
Membrane prepared by Nonsolvent-induced phase separation (NIPS). (**a**) Preparing membrane steps based on the NIPS process [16]. (**b**) Gas separation diagram [26]. (**c**) (**I**) Surface of membrane; (**II**) cross-section of ion-selective transportation; (**III**) performance comparison; and (**IV**) microstructure [19]. (**d**) Preparation and application of membrane [28].

Nonsolvent-induced phase separation (NIPS) produces asymmetric membranes with superior permselectivity by immersing a homogeneous polymer solution into a nonsolvent bath, where mutual solvent–nonsolvent diffusion triggers polymer solidification (Figure 2a) [15,16]. In the membrane fabrication process, water typically serves as the nonsolvent, whereas strong acids act as the primary solvents to obtain sulfonated PEEK membrane. Researchers have also focused on introducing crystallization-inhibiting functional groups to disrupt the ordered arrangement of PEEK polymer chains to enhance solubility and avoid the risk of strong acid corrosion.

Processing PEKK into asymmetric membranes via NIPS necessitates aggressive sulfonic acid solvents such as methanesulfonic acid (MSA), sulfuric acid (H_2_SO_4_), trifluoromethanesulfonic acid (TFMSA), or their mixtures, which alter chemical structure, reduce crystallinity, and consequently enhance solubility for PEEK materials [17,18]. Sulfonated PEEK (SPEEK) is the product obtained by the dissolution of strong sulfonic acid, which is commonly used to boost proton conductivity (Figure 2c) [19]. Notably, strict control over the degree of sulfonation (DS) is critical as it profoundly impacts the membrane solvent stability. Low DS (around 4 mol%) confers resistance to strong polar organic solvents (such as DMF, DMSO, DMAc, and pyridine), whereas higher DS (above 40 mol%) induces solubility in these solvents at room temperature [20]. PEEK even dissolves in hot water at 100% DS. This sulfonation-based approach offers a rapid, simple, and economical surface treatment process, allowing tailored regulation to meet specific requirements [21,22]. However, these sulfonic acid solvents induce aromatic ring sulfonation and main-chain scission, undermining solvent resistance and reducing mechanical strength. To address this, Dong et al. incorporated SPEEK and an ethylene glycol/water solvent into the poly (vinyl alcohol) hydrogel network to increase mechanical properties to achieve a strain of 663% and a tensile strength of 4.37 MPa [23].

Another widely adopted approach involves pre-modifying PEEK to enhance its solubility prior to fabricating films via the nonsolvent induced phase separation (NIPS) method. For example, A cardo-group-modified PEEK (PEEK-WC) retains the PEEK’s inherent superior thermal stability, mechanical strength, and chemical resistance while exhibiting markedly enhanced solubility in organic media [24]. The bulky cardo pendant group inhibits crystallization, enabling dissolution in chlorohydrocarbon solvents (such as chloroform) and polar organic solvents (including dimethyl formamide, N-methyl pyrrolidone, and tetrahydrofuran) [25]. PEEK-WC membranes fabricated via the NIPS are typically employed as gas separation membranes (Figure 2b), yet lower permeance restricts industrial applications [26,27]. Researchers often incorporate additives like poly (vinyl pyrrolidone) or ZIF-8 to regulate membrane structure, thereby tuning the gas permeance (Figure 2d) [28,29]. PEEK-WC could also be sulfonated to form SPEEK-WC [30]. Alternatively, Hendrix et al. developed additional modification strategies including copolymerization with alternative monomers and covalent crosslinking (Table 1) [31,32], further expanding the range of processable PEEK derivatives for NIPS. Furthermore, nonsolvent additives dosage and solvent–nonsolvent exchange rate should also be discussed in the future after ensuring solid powder dissolution, which will affect membrane structure and regulate membrane performance.

### 2.2. Thermally Induced Phase Separation (TIPS)

**Figure 3:**
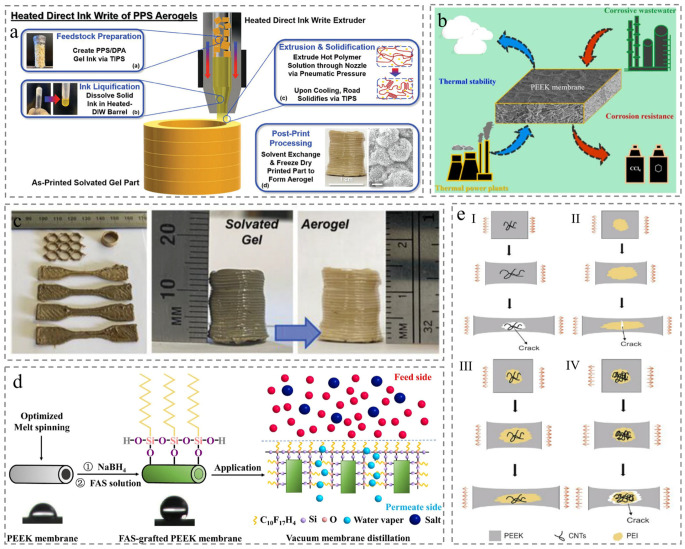
Membrane prepared by Thermally induced phase separation (TIPS). (**a**) Preparing membrane steps based on the TIPS process [34]. (**b**) Application and characteristics of prepared membranes [36]. (**c**) Electronic photos of materials of different shapes [34]. (**d**) Preparation, contact angle, and application of membrane [37]. (**e**) Schematic illustration of the deformation behavior for PEEK nanocomposites with (**I**) single PEEK; (**II**) PEEK and PEI; (**III**) PEEK, PEI, and low content CNTs; (**IV**) PEEK, PEI, and high content CNTs [40].

Thermally induced phase separation (TIPS) has emerged as the primary technique for fabricating porous PEEK membranes, overcoming the solubility constraints that impede conventional phase inversion methods (Figure 3a) [33,34]. This process enables macroscale tailoring of membrane geometry to satisfy specific design requirements and target applications (Figure 3c). Notably, TIPS method eliminates complex multistep processing and avoids environmental hazards and container corrosion associated with aggressive sulfonic acids (such as methanesulfonic acid, concentrated sulfuric acid, and trifluoromethanesulfonic acid), while preserving the intrinsic thermal stability and mechanical strength of PEEK membrane [35].

Diluents serve as a critical factor in regulating membrane structure during membrane preparation via the TIPS method. High-boiling-point diluents and plasticizers are typically used to dissolve PEEK material and then cast to fabricate membranes by a high-temperature extrusion process. However, despite progress in identifying suitable diluents for forming homogenous PEEK/diluent solutions, these solutions exhibit extremely low viscosity that isunable to sustain membrane formation. You et al. proposed incorporating amorphous polymer (such as polyetherimide, PEI) as a viscosity modifier in the PEEK blends to address this challenge. PEI is miscible with PEEK at high temperature and could be removed via solvents leaching during post-processing, yielding porous membranes. Membranes prepared via this approach exhibit excellent thermal stability and perform stably in emulsion separation involving corrosive components (Figure 3b) [36]. Building on this, Cao et al. developed nanoporous PEEK hollow fiber membranes utilizing PEEK/PEI blends via the TIPS, followed by chemical extraction of PEI. The membrane be further modified via fluoroalkylsilane (FAS) grafting to enhance properties such as anti-wetting property and salt rejection (Figure 3d) [37]. However, a key limitation arises that, unlike amorphous PEI, semicrystalline PEEK undergoes crystallization during membrane formation, resulting in some PEI trapped in steric confinement by ordered PEEK polymer chains during leaching. Residual PEI reduces membrane porosity, impairs pore connectivity, and increases mass transfer resistance, ultimately diminishing permeability and lowering the mechanical strength [38,39]. Wang et al. proposed utilizing polyetherimide (PEI) as a bridge to link PEEK with carbon nanotubes (CNTs). This CNT/PEI/PEEK nanocomposite enhances toughness while minimizing viscosity (Figure 3e) [40]. Apart from diluents, polymer concentration and cooling rate also require detailed investigation during the membrane formation, as they too influence porous membranes preparation.

### 2.3. Chemical-Induced Crystallization (CIC)

**Figure 4:**
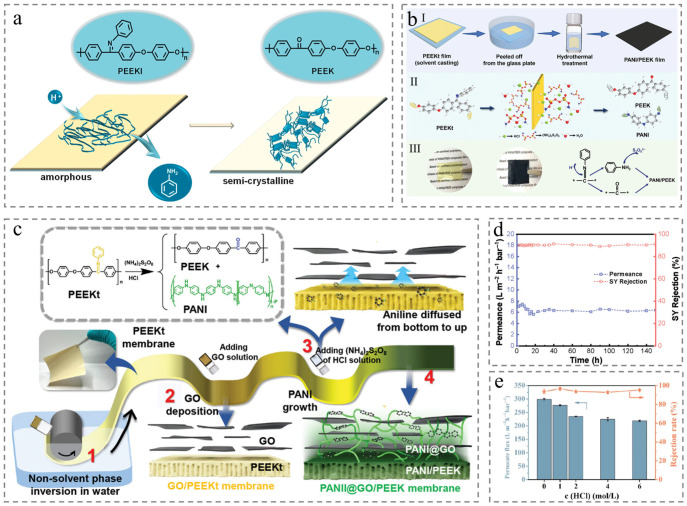
Membrane prepared by Chemical-induced crystallization (CIC) strategy. (**a**) Mechanism schematic diagram of the PEEKI transformed to the PEEK counterpart [43]. (**b**) Schematic illustration of (**I**) the membrane formation and (**II**)membrane reaction; (**III**) membrane image and reaction in membrane surface [47]. (**c**) Schematic illustration of the prepared membrane formation method and microstructure [48]. (**d**) The operation stability in organic solvent for 150 h [44]. (**e**) Water permeance and BSA removal efficiency of prepared membranes with long-term static immersion for 7 days in HCl from 1 M to 6 M [45].

While the TIPS method avoids the corrosion risk of strong acid, it suffers from suboptimal water permeance and separation performance. To address this, the chemical-induced crystallization (CIC) strategy has been developed, combining the typical NIPS process with a one-step heat treatment strategy, via forming the precursor polymer membrane followed by HCl heat treatment to achieve transformation [41]. Concretely, asymmetric precursor poly (etherether ketimine) (PEEKI) membrane is fabricated via the NIPS as amorphous PEEKI is soluble in common organic solvents (such as N-methyl pyrrolidone or N, N-dimethylacetamide) [42]. Subsequent HCl heat treatment converts PEEKI membrane to PEEK via acid-catalyzed Schiff base hydrolysis, where the ketimine functional groups in PEEKI are hydrolyzed to the carbonyl structures of PEEK (Figure 4a) [43]. This transformation reorganizes polymer chains from an amorphous to a semicrystalline state, overcoming traditional PEEK processing limitations while retaining the characteristic properties (thermal, chemical, and mechanical stability). Resultant membranes maintain structural integrity after 150 h of organic solvent filtration (Figure 4d) [44] or 168 h of hydrochloric acid solutions (Figure 4e) [45].

A key advantage of the CIC is its elimination of corrosive acids and extreme temperature conditions, enabling versatile PEEK membrane fabrication using common solvents [46]. Notably, strategies like confined polymerization (introducing additives during PEEK precursor hydrolysis) have addressed the PEEK interfacial incompatibility in separation membranes, facilitating the customization of solvent-resistant nanofiltration (SRNF) membranes. For instance, Jiang et al. blended the polyaniline (PANI) with PEEKI to overcome PEEK’s poor membrane-forming ability and PANI agglomerates (especially at >15% PANI content). By leveraging the co-growth of the two polymers in confined spaces (Figure 4b), PANI preferentially grows in the free volume of PEEK chains, inducing ordered matrix arrangements, yielding composite membranes with stable corrosion resistance, enhanced dielectric/conductive properties, and promising applications in electronic packaging [47]. Additionally, a novel strategy combining solvent-resistant PEEK membranes with ultrafast water transport of 2D nano-sheets has mitigated the permeance-selectivity trade-off, achieving superior solvent flux, excellent rejection, and exceptional stability in strong acids/alkalis with 100-h long-term durability (Figure 4c) [48]. However, it is necessary to investigate the crystallinity of PEEK in the process of PEEKI conversion to PEEK membrane because crystallinity influences the solvent resistance and is closely linked to the hydrochloric acid concentration and hydrolysis temperature in the hydrolysis process.

## 3. Hydrophilic Modifications

PEEK membrane with acid, alkali, and organic resistance is employed in diverse fields [49,50], yet PEEK is a semi-crystalline thermoplastic with a rigid aromatic backbone comprising alternating ether and ketone linkages, conferring high crystallinity and strong intermolecular interactions. Coupled with hydrophobic functional groups such as ester groups, this PEEK structure weakens the ability to accept hydrogen bonds and leads to inherently hydrophobic surfaces, facing critical barriers to practical application. During separation processes, these surfaces readily adsorb foulants (such as oils and organic macromolecules), triggering severe membrane fouling. This fouling not only causes rapid flux decline but also necessitates frequent cleaning and increased maintenance costs [51,52].

To address these issues, hydrophilic modification becomes imperative. The common hydrophilic modification is basically divided into two categories. One is to introduce hydrophilic modified functional groups to inhibit crystallinity and enhance the irregularity of the molecule. The other is to directly add hydrophilic substances. This paper classified hydrophilic modification through the order of implementing hydrophilic modification strategies, including hydrophilic modification before the formation of PEEK membrane, blending with hydrophilic substances during the formation of the membrane, and then hydrophilic modification after the formation of PEEK membrane (Table 2).

### 3.1. Pretreatment

Pretreatment strategies focused on hydrophilic modification of PEEK before membrane formation, offering advantages like reducing redundant post-processing and avoiding issues such as pore blocking or flux loss. These strategies primarily fall into two categories: molecular design to introduce hydrophilic groups into the polymer itself, and incorporation of hydrophilic additives during the conversion of PEEKI to PEEK membrane via the CIC method.

**Figure 5:**
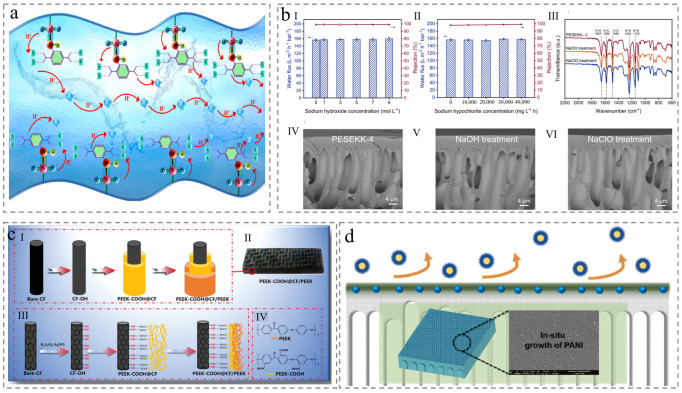
Membrane modified before membrane formation. (**a**) Mechanism schematic illustration of the ion transport pathway [54]. (**b**) Alkali and chlorine resistance demonstrated by performance and microstructure including (**I**) water permeance and (**II**) BSA removal efficiency after membrane immersion; (**III**) FTIR spectra before and after immersion; the cross-section of (**IV**) before immersion after immersion with (**V**) NaOH and (**VI**) NaClO [58]. (**c**) Schematic of the macroscopic process of CF/PEEK composite and molecular formula [55]. (**d**) Schematic diagram of antifouling mechanism [45].

The first category is molecular design, which enhances hydrophilicity by directly functionalizing PEEK with polar groups, eliminating the need for extraneous substances. For example, PEEK was sulfonated into SPEEK using concentrated sulfuric acid, which introduces hydrophilic sulfonic acid groups and is widely used to boost the proton conductivity in the batteries (Figure 5a) [53,54]. Similarly, carboxyl-modified PEEK (COOHPEEK) is synthesized via acylation followed by carboxylation, where abundant polar groups improve surface wettability (Figure 5c) [55]. Additionally, PEEK remains amenable to hydrophilic modification through surface functionalization with polar groups including hydroxyl (−OH) and nitro (−NO_2_) [56,57].

The second category involves incorporating hydrophilic additives during PEEKI-to-PEEK conversion. For instance, Jiang et al. introduced polyaniline (PANI) via in situ growth during acidolysis of PEEKI to PEEK for hydrophilic modification. The resulting membrane achieved a water permeance of 302 L m^−2^ h^−1^ bar^−1^ and a BSA rejection exceeding 97%, presenting superior permeability and excellent anti-fouling properties (Figure 5d) [45]. Similarly, Cai et al. introduced sulfone functional groups to develop a novel poly (ether sulfone ether ketone ketone) (PESEKK) with ultra-high flux, improving the hydrophilicity of PEEK while maintaining superior resistance to alkalis, chlorine, and organic solvents (Figure 5b) [58].

### 3.2. Co-Blending

Co-blending modification refers to the integration of base polymer with hydrophilic additives to form homogeneous polymer composites, which strategically tunes dope solution composition to mitigate inherent polymer limitations while enhancing material performance. This approach enables concurrent membrane formation while preserving structural integrity, offering an effective pathway for performance optimization [59].

**Figure 6:**
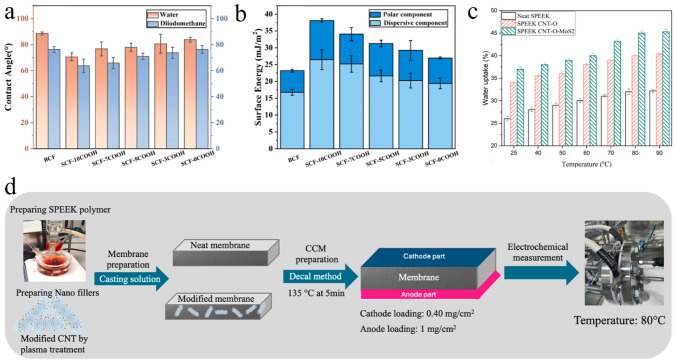
Membrane modified via co-blending. The (**a**) contact angle and (**b**) surface energy for different carbon fibers [60]. (**c**) Water uptake at different temperatures for the fabricated membrane [61]. (**d**) Schematic diagram of the membrane fabrication process and test conditions [61].

To engineer membranes with superior comprehensive performance, researchers typically select appropriate hydrophilic components for blending with PEEK, yielding membranes that retain PEEK’s inherent advantages (such as high-temperature resistance, chemical stability, and excellent mechanical strength) while incorporating the hydrophilic characteristics of the secondary component. For instance, Wang et al. blended carboxylated polyimide with PEEK, introducing carboxyl groups that reduced the water contact angle (Figure 6a) and increased the polar component of surface energy (Figure 6b), directly validating enhanced hydrophilicity. Notably, this modification also improved mechanical properties of the membrane, with flexural strength and modulus increasing by 15.8% and 28.5%, respectively, demonstrating the ability of co-blending to simultaneously enhance both hydrophilicity and mechanical properties [60]. Similarly, Testi et al. compared the water uptake of sulfonated PEEK (SPEEK) with that of SPEEK/CNT-O-MoS_2_ composite membranes, where CNT-O-MoS_2_ nanofillers were incorporated into the SPEEK matrix (Figure 6d). At the same temperature, the composite membrane reached 45.3% water uptake that significantly higher than 32.2% of pure SPEEK (Figure 6c) [61]. This improvement was ascribed to the presence of Mo-based structures and sulfonic groups promoting water molecule mobility within the modified membrane matrix, highlighting that co-blending with functional nanofillers could outperform single-component modifications (such as pure sulfonation) in enhancing hydrophilicity.

### 3.3. Post-Treatment

Post-treatment strategies enhance the hydrophilicity by introducing hydrophilic moieties directly onto the PEEK membrane surface, primarily through two approaches: surface grafting/coating and the introduction of polar functional groups/hydrophilic substances after membrane formation [62].

**Table 2:** Summary table of hydrophilic modification methods.

Hydrophilic Modification Methods	Examples	Water Contact Angle	Reference
Pretreatment	Graft sulfonic acid group	From 110° to 73°	[53,54]
	Grafted carboxyl group	From 111° to 24°	[55]
	Incorporating polyaniline	From 109° to 78°	[45]
Co-blending modification	Blend with carboxylated polyimide	From 89° to 70°	[60]
	Blend with carbon nanotube and MoS_2_	From 80° to 65°	[61]
Post-treatment	Grafting ethyl hydroxy acrylate	From 97° to 30°	[63]
	Grafting polyallylamine	From 91° to 75°	[64]

**Figure 7:**
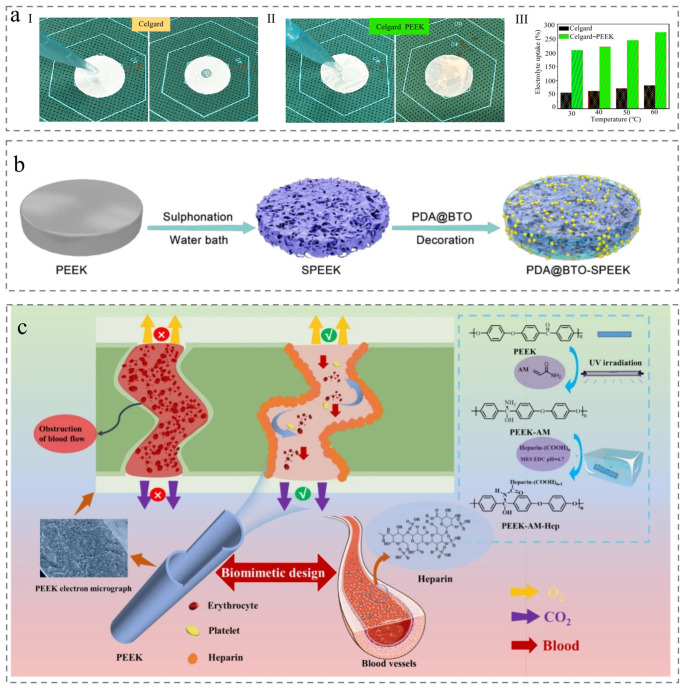
Membrane modified after membrane formation. (**a**) (**I**) Electrolyte uptake; (**II**) electrolyte wettability; and (**III**) electrolyte contact angle measurements [67]. (**b**) Schematic illustration of the preparation process [69]. (**c**) Membrane preparation and membrane application diagram [65].

The first category is surface grafting and coating on the PEEK membrane surface. For example, Huang et al. enhanced PEEK membrane performance by grafting hydrophilic ethyl hydroxy acrylate (HEA) via UV irradiation. Through systematic investigation of grafting degree effects, they demonstrated that optimized grafting significantly improved membrane permeability and antifouling capabilities [63]. Similarly, Hendrix et al. introduced hydrophilic polyallylamine as a crosslinker into pristine PEEK membranes, introducing free amine groups to modulate membrane structure and enhance organic solvents permeance [64]. Liu et al. further demonstrated this strategy by grafting heparin and heparin-like substances onto PEEK surfaces via UV irradiation grafting and amidation condensation reactions, achieving enhanced gas permeability (Figure 7c) [65]

Beyond surface grafting, post-treatment strategies also include the introduction of polar functional groups and hydrophilic substances after membrane formation [66]. For example, sulfonating PEEK membranes by incorporating polar groups (SO_3_H) directly improves surface wettability (Figure 7a) [67]. Notably, sulfonation can serve as a foundation for further hydrophilic modification [68]. For instance, Cheng et al. introduced additional hydrophilic substances (hydrophilic polydopamine) for secondary modification after sulfonating PEEK to enhance hydrophilicity and create a porous structure (Figure 7b) [69]. Additionally, alternative methods convert surface carbonyl groups to hydroxyl groups (via wet chemical reduction or UV-assisted processes), yielding hydroxylated PEEK (PEEK-OH) with improved hydrophilicity [70,71].

## 4. Applications for Water Remediation

Membrane technology with energy-efficient separations encounters increasingly complex environments due to industrialization and resource constraints, where material performance limitations impede practical implementation. Crucially, conventional polymers such as polyethersulfone (PES), polyetherimide (PEI), and polyacrylonitrile (PAN) failed to sustain durability under combined organic solvents, acids, alkalis, high pressure, and elevated temperatures without chemical modification. By contrast, intrinsically PEEK delivers exceptional stability without crosslinking, exhibiting chemical resilience (resistant to pH 1–14 and organic solvents), thermal tolerance (retaining properties up to 250 °C), and mechanical robustness (tensile strength of 90–100 MPa) [72]. This inherent stability enables PEEK and its derivatives to outperform other polymers in severe separation environments.

### 4.1. Synthetic Dyes Removal

Synthetic dyes are extensively used in textiles, paper, plastics, leather, and cosmetics industries, generating massive dye-contaminated wastewater that threatens human health and the ecosystems. Conventional membrane materials (like polyethersulfone, polyimide, polyacrylonitrile, polylactic acid) undergo irreversible structural dissolution and performance decrease through pore collapse and polymer chain scission when exposed to dye wastewater with strong acid, strong alkali, limiting their practical application in a harsh environment [73,74].

**Figure 8:**
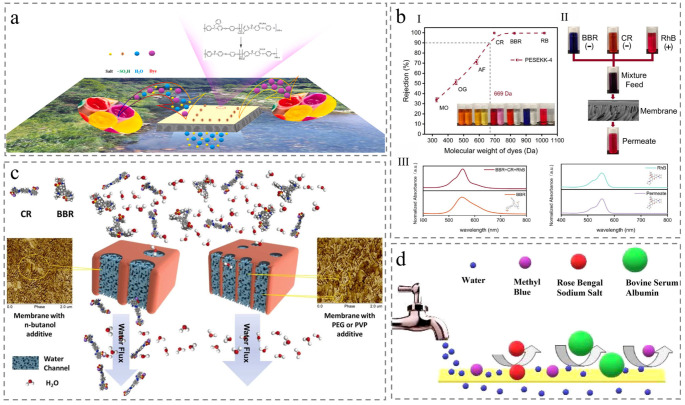
Membrane application for removing synthetic dyes. (**a**) Preparation and application of membrane [76]. (**b**) Dye removal performance and selective separation performance, including (**I**) separation behavior for dye with different molecular weights, (**II**) a process of multi-component dye filtration, and (**III**) results of UV-vis absorption spectra [59]. (**c**) Comparison of dye separation process for different membranes [77]. (**d**) Membrane separation process of different dye types [74].

In response, recent research has focused on developing chemical-resistant and durable ultra-permeable membranes capable of separating small organic molecules (like inorganic salt or dye) in complex textile wastewater. Some researchers showed PEEK membrane performance stability when exposed to acid solutions (2, 4, 6, and 8 M hydrochloric acid) for 100 h and organic solvents (n-methyl pyrrolidone, Ethanol, and tetrahydrofuran) for 120 h, exhibiting perfect potential in chemical resistance while maintaining durable performance [75]. For instance, Cao et al. developed a modified functional PEEK membrane that achieved exceptional selectivity (salt rejection <10%, dye rejection 98.8%) with superior flux (34 L m^−2^ h^−1^ bar^−1^). Notably, the membrane performance remained stable over 10 cycles with robust resistance to organic solvents and strong antifouling properties, underscoring its promising potential for sustainable textile wastewater treatment (Figure 8a) [76]. To clarify key factors governing membrane performance, Jiang et al. investigated the pore size effect on the selectivity and permeability toward Rose Bengal sodium salt (RB, 1017 Da), Methyl Blue (MB, 799 Da), and Bovine serum albumin (BSA, 66.4 K Da). They emphasized the persistent permeance-selectivity trade-off in membrane design, proposing that balancing the permeance of porous membranes with the superior selectivity of dense membranes could broaden applications in separation and purification processes (Figure 8d) [75]. Complementing these insights, Pan et al. optimized membrane structure by increasing polymer concentration on membrane surface, accelerating phase separation during the NIPS process. This strategy yielded a thinner, denser skin layer, concurrently enhancing permeability and dye separation performance (Figure 8c) [77]. In addition to the mechanism of pore size sieving above, the mechanism of the Donnan effect also plays a significant role in dye rejection. Cai et al. fabricated high-performance loose nanofiltration (NF) membranes capable of precisely separating negatively and positively charged dyes in mixed solutions, further expanding the scope of membrane-based dye separation (Figure 8b) [59]. However, given the variations in dye structures, molecular sizes, and aggregation degrees, practical applications require designing distinct separation processes based on the specific components of dye wastewater. Researchers could integrate membranes with different pore sizes or different charges to maximize the advantages of each separation unit and enable the efficient removal of harmful components from dye wastewater while facilitating the reuse of valuable resources such as dyes and water.

### 4.2. Organic Solvent Nanofiltration

Organic solvent nanofiltration (OSN) technology designed for solute separation and solvent recovery in organic systems has advanced rapidly to mitigate the significant energy and environmental burdens of separation processes in organic solvents [78,79,80]. Renowned for high efficiency and low energy consumption, OSN is widely applied in non-aqueous or organic solvent-water mixed systems, spanning organic synthesis, petrochemicals, pharmaceutical manufacturing, and food processing [81,82]. However, traditional polymer membranes such as polyethersulfone (PES) and polysulfone (PSF) often swell or even dissolve in organic solvents, severely limiting their viability in OSN applications [83].

**Figure 9:**
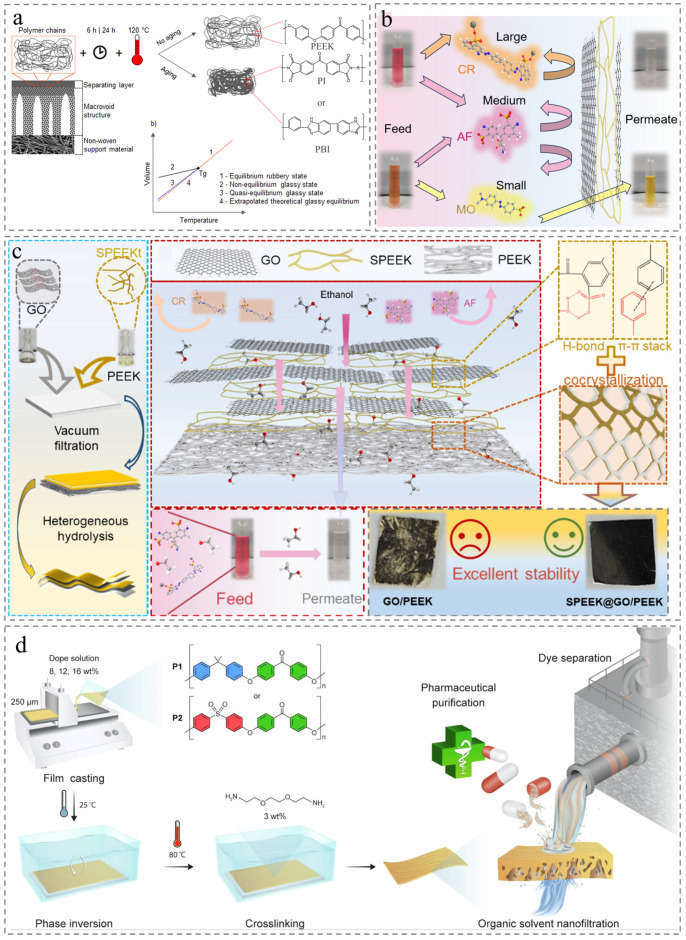
Membrane application for organic solvent nanofiltration. (**a**) Schematic of time and temperature effect on the polymer chains and qualitative volume-temperature curves for glass-forming polymers [84]. (**b**) The process of separating various dyes [86]. (**c**) Diagram including preparation method, separation mechanism, and membrane characteristics [86]. (**d**) Schematic overview of membrane preparation via phase inversion, followed by membrane crosslinking, and the applications in dye and pharmaceutical purification [87].

Against this backdrop, PEEK membranes have emerged as a promising alternative. Burgal et al. observed that PEEK demonstrates exceptional aging resistance under extreme conditions including 120 °C high-temperature filtration and air annealing with aggressive solvents. This resilience contrasts sharply with the substantial degradation of crosslinked polyimide (PI) and polybenzimidazole (PBI) membranes, which suffer from permeance loss and embrittlement (Figure 9a) [84]. This highlights semi-crystalline PEEK as an attractive OSN candidate due to superior solvent resistance and thermal stability. However, PEEK membranes face limitations of low permeability and poor separation efficiency, even after N, N-dimethylformamide (DMF) activation to enhance membrane selectivity, hindering their OSN application [43]. To address this, researchers have developed various tailored modifications. For example, Zhang et al. fabricated defect-free polyesteramide (PEA) nanofilms in situ on PEEK surfaces, enabling rapid solvent transport and exceptional OSN separation performance [85]. Similarly, Peng et al. developed a composite membrane via a weaving strategy that uses PEEK as a base and sulfonated PEEK (SPEEK) as a “linker” to connect graphene oxide (GO) fragments (promising OSN material because of their oxygen-containing functional groups) (Figure 9c). The prepared membrane showed excellent organic solvents permeance (such as 121.77 L m^−2^ h^−1^ bar^−1^ for acetone and 22.71 L m^−2^ h^−1^ bar^−1^ for DMF) and high separation efficiency for Acid fuchsin (AF, 585 Da) exceeding 92% in DMF, coupled with superior stability (Figure 9b) [86]. Additionally, researchers incorporated sulfonyl (SO_2_) or methyl (–CH_3_) groups into the PEEK polymer backbone and crosslinked with 2,2′-(ethylenedioxy) bis(ethylamine) (EDBEA) to explore the potential effect of polar and nonpolar functional groups on membrane properties and OSN separation performance (Figure 9d) [87,88]. However, current PEEK-based OSN technology is limited to treating pollutants such as dyes and pharmaceuticals. Researchers could attempt to incorporate materials such as covalent organic frameworks (COFs) into PEEK membranes, and the COF nanoparticles can migrate within the intermediate layers of organic solvents to form highly selective sub-nanometer pores, expanding the application in industrial wastewater treatment.

### 4.3. Natural Organic Matter Removal

Membrane separation technology is widely employed for contaminated water, yet it faces significant membrane fouling problems primarily caused by aquatic natural organic matter (NOM, which includes by-products of plant decomposition and microbial metabolic activity) [89]. NOM accumulation on membrane surfaces forms cake layers, causing flux decline, compromised water quality, and elevated energy consumption. The intrinsic hydrophobicity of PEEK exacerbates non-specific adsorption of proteins and NOM, restricting their application for natural organic matter removal. Enhancing surface hydrophilicity remains a proven strategy to mitigate such fouling.

**Figure 10:**
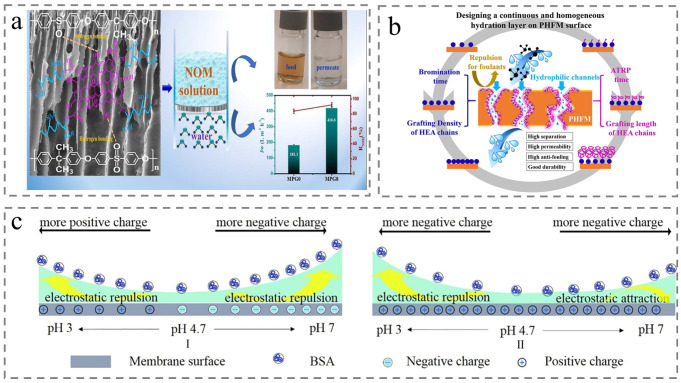
Membrane application for natural organic matter. (**a**) Preparation, characterization, and application of the prepared membrane [90]. (**b**) The schematic diagram includes condition exploration, separation mechanism, and membrane characteristics 91]. (**c**) Diagram of the electrostatic interaction between the BSA and the membrane surface under different pH values with (**I**) unmodified membrane; (**II**) modified membrane [92].

Kumar et al. fabricated hydroxylated SPEEK grafted graphene oxide (SPK-g-GO) modified membrane, which exhibited significant NOM resistance (Flux Recovery Ratio = 94.5%) and high NOM separation efficiency (92%). These results verified a negative correlation between hydrophilicity and membrane fouling (Figure 10a) [90]. Notably, the hydrophilicity degree of the membrane surface also influences fouling inhibition. Liu et al. constructed hydrophilic 2-hydroxylethyl acrylate (HEA) chains on PEEK membrane surface, modulating grafting density and grafting length of the HEA to simultaneously enhance the permeance, separation efficiency, and anti-fouling performance of the membrane (Figure 10b) [91]. Beyond hydrophilicity, membrane surface charge also significantly affects NOM removal efficiency and antifouling performance. Jiang et al. grafted neutral hydroxyethyl acrylate (HEA, neutral), negatively charged acrylic acid (AA), and positively charged acryloxyethyl trimethylammonium chloride (DAC) onto PEEK membranes, respectively, yielding membranes tailored for NOM with different charge properties. Additionally, they investigated the impact of pH on membrane fouling behavior (Figure 10c) [92]. Furthermore, researchers could develop responsive membranes to expand upon the treated range of natural organic matter.

### 4.4. Oil–Water Separation

Oil–water separation has become an urgent challenge amid escalating water pollution, driven by large-scale industrial wastewater discharge and frequent oil spill incidents. Membrane separation technology is widely recognized as one of the most effective strategies, offering advantages like low cost, low energy consumption, operational simplicity, and high selectivity [93]. While polymers (such as polylactate, polyacrylonitrile, and polyvinyl alcohol) are commonly used as membrane materials due to their tunable physicochemical properties, they suffer from inadequate mechanical strength, poor corrosion resistance and thermal instability, limiting practical implementation. In contrast, PEEK membranes stand out as a promising alternative featuring corrosion resistance, and thermal stability.

**Figure 11:**
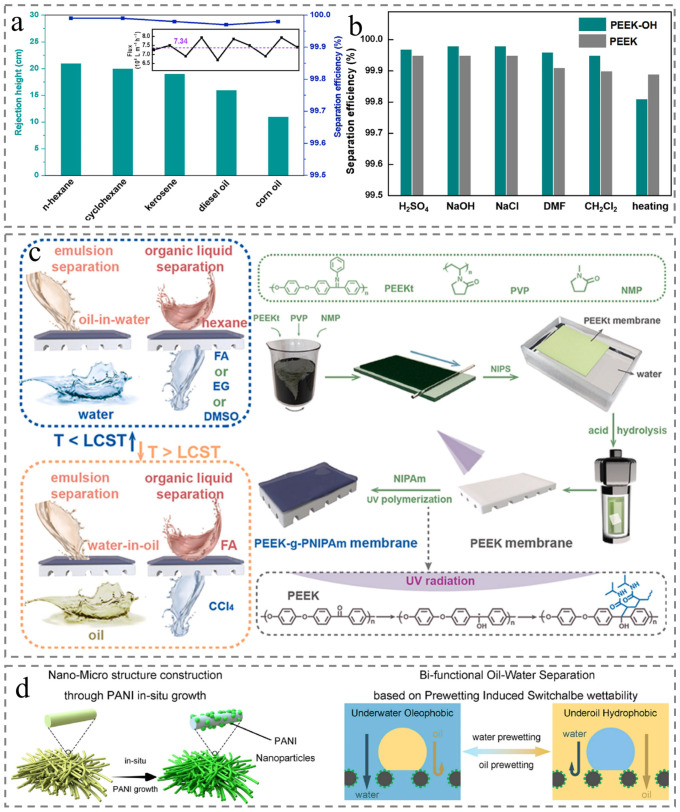
Membrane application for oil–water separation. (**a**) The rejection heights and removal efficiencies for different oils [94]. (**b**) The separation efficiencies after immersion in H_2_SO_4_, NaOH, NaCl, DMF and dichloromethane for 500 h and being heated at 200 °C [94]. (**c**) Schematic diagram of preparation process and oil–water separation process [95]. (**d**) Nano-micro structure construction and Bi-functional oil–water separation [96].

Building on these inherent advantages, Wang et al. fabricated PEEK textile via melt spinning, followed by reduction to form PEEK-OH textile. The membrane achieved high separation efficiency for various oils (Figure 11a) and maintained stable performance even after 500 h of immersion in strong acids, strong bases, and organic solvents (Figure 11b), highlighting PEEK’s potential as a robust material for oil–water separation [94]. To further enhance functionality, researchers have explored stimulus-responsive modifications. For instance, Yang et al. grafted thermo-responsive poly (N-isopropylacrylamide) (PNIPAm) onto PEEK membranes via UV-induced polymerization. The resulting PEEK-g-PNIPAm membranes displayed temperature-tunable wettability: superhydrophilicity (water contact angle = 0°) at 25 °C and hydrophobicity (water contact angle = 100°) at 40 °C. This thermo-responsiveness enabled efficient separation of diverse oil-bearing systems with rejection exceeding 99.0% by leveraging temperature-dependent wettability switching (Figure 11c) [95]. Beyond temperature responsiveness, dual-functional surface engineering offers another route to switchable separation. Zhao et al. prepared PEEK/PANI bifunctional membranes combined with in situ polyaniline (PANI) growth, which exhibit switchable underwater oleophobicity and underoil hydrophobicity. Their separation process is regulated by prewetting liquid: when prewetted with water, the membrane allows gravity-driven water permeation (retaining light oil) from light oil–water mixtures; when prewetted with organic solvents, it enables gravity-driven oil permeation (retaining water) from water-heavy oil mixtures (Figure 11d) [96]. However, the flexible polymer membranes are easily contaminated by oil in the long-term cyclic operation. Thus, while enhancing membrane hydrophilicity to improve antifouling performance, attention could be directed toward incorporating rigid particles. These particles may facilitate the demulsification of oil-in-water emulsions and extend the membrane life.

## 5. Conclusions

Membrane technology has emerged as a prominent green solution in recent decades, yet conventional materials suffer from insufficient chemical/thermal stability and mechanical strength, limiting their efficacy in harsh or complex environments. Polyetheretherketone (PEEK) stands out by integrating ceramic-like chemical and thermal stability with polymer-derived flexibility and cost-effectiveness, complemented by superior chemical resistance, exceptional mechanical strength, and outstanding thermal stability. These attributes make PEEK a highly promising candidate for wastewater treatment under harsh or complex conditions. Notably, PEEK applications in dye separation, oil–water separation, organic solvent recovery, and natural macromolecule removal have advanced toward practical implementation. This paper discusses three PEEK membrane fabrication methods, highlights hydrophilic modification strategies to enhance flux and fouling resistance in water treatment, and reviews current PEEK membrane applications in the wastewater treatment field. Cumulative research demonstrates that functionalized PEEK plays a pivotal role in water treatment.

## 6. Outlooks

Despite these advancements, several critical challenges remain:

Firstly, inadequate research has been conducted on PEEK membrane structure tailoring. Although the previous works proved that the combination of PEEK and other materials is effective with solvent resistance and high mechanical strength in water treatment, the interface of the heterostructure between PEEK and other materials should be considered to ensure long-term stability [97]. And try to use degradable or high-stability materials for these adopted modified materials, avoiding secondary pollution in the water treatment process.

Secondly, although hydrophilic modified PEEK membranes could partially alleviate membrane fouling under laboratory conditions, the membrane surface deposition of diverse microorganisms and pollutants (like nanoplastics and natural organic matter) inevitably exacerbates membrane fouling, resulting in decreased water permeance while treating actual water [98]. Thus, it is necessary to explore more regular findings of focus on various real water parameters, offering valuable guidance for both material development and practical applications.

Third, the development of modification techniques to expand novel wastewater treatment scenarios (like seawater desalination, nanoplastic removal, and synthetic organic) and wastewater recycling (like lithium magnesium extraction and mining in mine wastewater), because PEEK has economic benefits in high temperature and strong acid and alkali environments with superior intrinsic mechanical and thermal stability.

Furthermore, the incorporation of machine learning will play a significant role in membrane design areas [99]. Machine learning could carefully integrate data sets through algorithms, predicting critical membrane performance (such as permeance and selectivity) and accurately locating materials with great potential in different water treatment directions [100]. In addition, machine learning is also utilized to design the monomer structure, establish a quantitative structure-performance relationship, and control the crystallinity to improve the solubility without damaging the organic solvent resistance. Therefore, it is necessary to integrate the use of PEEK membranes with machine learning to provide a multi-functional solution for the preparation and application of membranes.

In summary, PEEK offers a robust platform to enhance chemical/thermal stability and mechanical strength in water treatment materials. While progress has been made in PEEK membrane research, addressing the aforementioned challenges is pivotal to accelerating their practical deployment in water treatment.

## Figures and Tables

**Table 1:** Reported pure and modified PEEK polymers.

Polymer	Abbreviation	Molecular Structure
Poly (etherether ketone)	PEEK	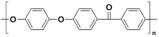
Poly(oxa-p-phenylene-3,3-phtalido-p-phenylenxoxa-p-phenylenoxoxy-p-phenylene) with Cardo group	PEEK-WC	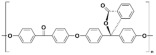
Sulphonated poly (etherether ketone)	SPEEK	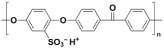
Sulphonated poly(oxa-p-phenylene-3,3-phtalido-p-phenylenxoxa-p-phenylenoxoxy-p-phenylene) with Cardo group	SPEEK-WC	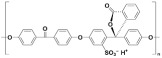
Modified PEEK with diphenolic acid	VAPEEK	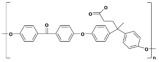
Modified PEEK with tertiarybutylhydroquinone (TBHQ) (instead of hydroquinone)	TBPEEK